# Acute effects of cochleostomy and electrode-array insertion on compound action potentials in normal-hearing guinea pigs

**DOI:** 10.3389/fnins.2023.978230

**Published:** 2023-02-08

**Authors:** Saad Jwair, Dyan Ramekers, Hans G. X. M. Thomeer, Huib Versnel

**Affiliations:** ^1^Department of Otorhinolaryngology and Head and Neck Surgery, University Medical Center Utrecht, Utrecht University, Utrecht, Netherlands; ^2^UMC Utrecht Brain Center, Utrecht University, Utrecht, Netherlands

**Keywords:** electrocochleography, electrode array, cochleostomy, compound action potential, cochlear microphonics, cochlear implantation, osseous spiral lamina

## Abstract

**Introduction:**

Electrocochleography (ECochG) is increasingly used in cochlear implant (CI) surgery, in order to monitor the effect of insertion of the electrode array aiming to preserve residual hearing. However, obtained results are often difficult to interpret. Here we aim to relate changes in ECochG responses to acute trauma induced by different stages of cochlear implantation by performing ECochG at multiple time points during the procedure in normal-hearing guinea pigs.

**Materials and methods:**

Eleven normal-hearing guinea pigs received a gold-ball electrode that was fixed in the round-window niche. ECochG recordings were performed during the four steps of cochlear implantation using the gold-ball electrode: (1) Bullostomy to expose the round window, (2) hand-drilling of 0.5–0.6 mm cochleostomy in the basal turn near the round window, (3) insertion of a short flexible electrode array, and (4) withdrawal of electrode array. Acoustical stimuli were tones varying in frequency (0.25–16 kHz) and sound level. The ECochG signal was primarily analyzed in terms of threshold, amplitude, and latency of the compound action potential (CAP). Midmodiolar sections of the implanted cochleas were analyzed in terms of trauma to hair cells, modiolar wall, osseous spiral lamina (OSL) and lateral wall.

**Results:**

Animals were assigned to cochlear trauma categories: minimal (*n* = 3), moderate (*n* = 5), or severe (*n* = 3). After cochleostomy and array insertion, CAP threshold shifts increased with trauma severity. At each stage a threshold shift at high frequencies (4–16 kHz) was accompanied with a threshold shift at low frequencies (0.25–2 kHz) that was 10–20 dB smaller. Withdrawal of the array led to a further worsening of responses, which probably indicates that insertion and removal trauma affected the responses rather than the mere presence of the array. In two instances, CAP threshold shifts were considerably larger than threshold shifts of cochlear microphonics, which could be explained by neural damage due to OSL fracture. A change in amplitudes at high sound levels was strongly correlated with threshold shifts, which is relevant for clinical ECochG performed at one sound level.

**Conclusion:**

Basal trauma caused by cochleostomy and/or array insertion should be minimized in order to preserve the low-frequency residual hearing of CI recipients.

## 1. Introduction

Cochlear implants (CIs) have been tremendously successful in restoring speech perception in severely hearing impaired patients (Carlson, 2020). The CI converts sound into electrical current pulses that stimulate the auditory nerve, thereby bypassing affected and degenerated hair cells. However, for most CI recipients, speech perception is suboptimal and requires considerable listening effort, especially in situations with background noise (Gifford and Revit, 2010). Residual hearing, i.e., threshold < 80 dB hearing level at 125–500 Hz, is present in around 50% of CI recipients (Kant et al., 2022a), and can be used to improve speech perception, e.g., with use of electro-acoustical stimulation (Gstoettner et al., 2004; Dhanasingh and Hochmair, 2021). Preservation of residual hearing after cochlear implantation has been reported by several studies (see for reviews Miranda et al., 2014; Snels et al., 2019). However, Kant et al. (2022a) have shown that in one CI center residual hearing was (partially) lost in most CI recipients (90%) 3 months after implantation.

Residual hearing can be acutely affected by cochlear implantation in several ways. The cochlear structures can be directly damaged by insertion of the electrode array, such as with scalar translocation of the array (Jwair et al., 2021). In addition, the basal cochlear turn can also be damaged by the drill that is used for surgically approaching the cochlea (Richard et al., 2012). Mechanical trauma to hair cells and auditory nerve fibers by drilling and/or array insertion directly impacts the residual hearing. In addition, trauma to cochlear structures can lead to mixture of endolymph, located in the cochlear duct, and perilymph, which is located in the scala tympani and scala vestibuli. This mixture abolishes the endocochlear potential (Reiss et al., 2015). Acute structural trauma might also alter the mechanics of the basilar membrane, impeding the traveling wave, thereby potentially impacting cochlear areas located more apically to the site of trauma. Residual hearing can also deteriorate by sudden changes in intra-scalar pressure (Gonzalez et al., 2020), blood and bone dust entering the cochlea (Radeloff et al., 2007), and noise-related trauma associated with drilling of the bone (Pau et al., 2007).

It is clear that electrocochleography (ECochG) has the potential to detect physiological changes and trauma intracochlearly (Giardina et al., 2019). ECochG has emerged as a promising tool that might aid the surgeon in minimizing acute trauma, thereby preserving residual hearing of CI patients (Bester et al., 2017). ECochG refers to the recording of electrical potentials generated by hair cells and auditory nerve in response to acoustic stimuli. ECochG research has been performed since the sixties to assess cochlear pathologies such as endolymphatic hydrops in Ménière’s disease (Eggermont, 2017). The resurgence of research regarding ECochG is linked to relatively new ability to record cochlear potentials using the intracochlear electrode array (Calloway et al., 2014; Bester et al., 2017). ECochG can provide feedback about the cochlear structures during electrode insertion, based on which the surgeon can adapt the insertion to potentially reduce trauma (Weder et al., 2020). In addition, ECochG can shed light on which aspects of cochlear implant surgery are detrimental for hearing preservation (Weder et al., 2021; Lenarz et al., 2022).

Currently, however, ECochG responses during cochlear implantations show large variability. This variability can be caused by several factors, such as trauma to cochlear structures, physiological changes without trauma, and due to movement of the electrode during insertion (Dalbert et al., 2021). Often there is a discrepancy seen between intraoperative ECochG responses and postoperative audiometric thresholds in CI recipients, probably due to this large variability (Adunka et al., 2016).

To understand the ECochG better during cochlear implantation, several animal studies investigated the relationship between ECochG and acute trauma in normal-hearing and noise-induced hearing loss gerbils and guinea pigs. Smaller compound action potentials (CAP) and cochlear microphonics (CM) responses were seen after electrode insertion. In addition, even though small responses were in most cases associated with histological trauma, some cases showed no association with histological trauma, i.e., to osseous spiral lamina (OSL), basilar membrane, spiral ligament (Choudhury et al., 2011, 2014; DeMason et al., 2012; Honeder et al., 2016, 2019). In addition, ECochG responses to low frequencies (associated to the apical cochlear turn) can be affected by basal trauma such as OSL and basilar membrane damage (Choudhury et al., 2011, 2014; Smeds et al., 2015), although electrode insertion was not affecting low frequencies in some studies (Robertson and Irvine, 1989; Chambers et al., 2019; Andrade et al., 2022). A recent study in CI recipients showed that insertion of a short electrode array can preserve the ECochG responses to the lower frequencies, indicating that basal trauma is not necessarily affecting apical areas (Dalbert et al., 2021). To our knowledge, just one study has described ECochG results after solely cochleostomy, i.e., without electrode insertion (Andrade et al., 2022). They recorded CAPs for frequencies between 2 and 32 kHz, and they found that cochleostomy did not affect the responses, whereas array insertion caused threshold shifts of around 20 dB at higher frequencies.

In the current study we investigated the degree to which cochlear potentials, in terms of primarily CAP thresholds, amplitudes and latencies, are affected by acute trauma during separate stages of the cochlear implantation procedure, i.e., cochleostomy and array insertion. We conducted ECochG at the round window (RW) varying stimulus frequencies from 250 Hz to 16 kHz in normal-hearing guinea pigs in order to be able to detect effects of trauma to both high and low frequencies. Cochlear implantation was performed with flexible electrode arrays (similar to those in humans). Histological analysis of the cochlea was conducted after the ECochG experiments, allowing for a thorough analysis of cochlear structures (including hair cell counts). Subsequently, ECochG responses were evaluated in relation to cochlear structural trauma.

## 2. Materials and methods

### 2.1. Animals and experimental design

**FIGURE 1:**
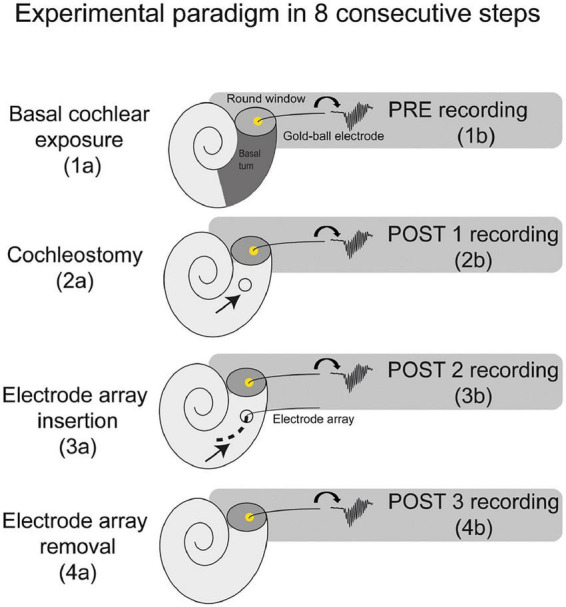
A schematic overview of the experimental paradigm. The electrocochleography was performed using the gold-ball electrode in the round window niche. The experiments consisted of 8 consecutive steps, with electrocochleography at 4 separate stages of the procedure, i.e., at PRE (before cochleostomy), POST1 (after cochleostomy), POST2 (after electrode-array insertion), and POST3 (after electrode-array removal).

Thirteen female albino guinea pigs (Dunkin Hartley; Hsd Poc:DH; ∼350 g) were obtained from Envigo (Horst, Netherlands) and kept under standard laboratory conditions (food and water *ad libitum*; lights on between 7:00 a.m. and 7:00 p.m.; temperature 21°C; humidity 60%). The same procedures were followed for all animals. ECochG was performed at four separate stages of surgery: before cochleostomy (PRE), after cochleostomy (POST1), after CI insertion (POST2), and after CI withdrawal (POST3), see Figure 1. In all four stages ECochG was performed with a custom-made gold-ball electrode that was fixated in the round window niche.

All surgical and experimental procedures were approved by the Animal Experiments Committee of Utrecht University (4315-1-01) and the Central Authority for Scientific Procedures on Animals (AVD1150020174315).

### 2.2. Surgical procedures

The animals were anesthetized by intramuscular injection of dexmedetomidine (Dexdomitor; Vetoquinol, Breda, Netherlands; 0.13 mg/kg) and ketamine (Narketan; Vetoquinol, Breda, Netherlands; 20 mg/kg). The animals were tracheostomized, and artificially ventilated with 1–2% isoflurane in O2 and N2O (1:2) throughout the experiment. Subsequently, needle electrodes were used for recordings of auditory brainstem responses (ABRs), with the active electrode placed subcutaneously behind the right ear, and the reference electrode subcutaneously at the midline of the frontal skull. The skull and the neck muscles overlying the bony bulla were exposed with one surgical incision along a line from the anterior medial side of the skull to retro-auricular right-ear region. One transcranial screw was placed on the skull, 1 cm anterior from bregma (ECochG reference electrode). After pushing the neck muscles aside, a bullostomy was performed to expose the right basal turn of the cochlea (PRE). To perform ECochG, a gold-ball electrode was used which consisted an isolated stainless steel wire (diameter 0.175 mm; Advent, Halesworth, United Kingdom) with a 0.5 mm diameter gold-ball micro-welded to the tip (Unitek 80 F, Unitek Equipment, Monrovia, CA, United States). The steel wire was bent about 90° at 2–3 mm from the gold-ball tip, which then was positioned in the RW niche, and the steel wire was subsequently fixed with an electrode holder (Versnel et al., 2007). Subsequently, a cochleostomy was manually performed with a 0.5 mm hand drill, just below (∼0.5 mm) the round window (POST1). This method has been previously performed without causing noticeable threshold shifts and/or hair cell loss (Ramekers et al., 2015, 2022). After the cochleostomy, a custom-made electrode array (Advanced Bionics; diameter 0.5 mm, length basal electrode to tip 3.5 mm, inter-electrode distance 1.0 mm) was inserted ∼4 mm into scala tympani (POST2) with all 4 electrodes of the array positioned intracochlearly. The diameter of the scala tympani at 5 mm from the round window is about 0.5 mm (Wysocki and Sharifi, 2005), which allows for the insertion depth of 4 mm. Lastly, the electrode array was removed (POST3). The intervals between the stages were approximately 1 to 2 h: between bullostomy and cochleostomy approximately 2 h, and both between cochleostomy and array insertion, and between array insertion and array removal approximately 1 h.

### 2.3. Electrophysiology

#### 2.3.1. Auditory brainstem response

After tracheostomy the ABRs were recorded using subcutaneously positioned needle electrodes (active electrode behind the right pinna; reference electrode on the skull, rostral to the brain on the midline; ground electrode in left hind limb). Broadband acoustic clicks (20 μs monophasic rectangular pulses; inter-stimulus interval 99 ms) were synthesized and attenuated using a TDT3 system (Multi-I/O processor RZ6; Tucker-Davis Technologies, Alachua, FL, United States), and presented in free field using a Bowers and Wilkens speaker (CCM683; 8 Ω; 25–130 W) at 10 cm distance from the right ear.

The signal was pre-amplified using a Princeton Applied Research (Oak Ridge, TN, United States) 5113 pre-amplifier (amplification × 5000; band pass filter 0.1–10 kHz). The amplified signal was digitized by the same TDT3 system for analysis (100 kHz sampling rate, 24-bit sigma-delta converter). The responses were averaged over 500 repetitions (maximum) and stored on a PC for offline analysis with custom MATLAB software. The sound level was attenuated in 10 dB steps, starting with maximum sound level at approximately 105 dB peak equivalent SPL (average of maximum sound level of 2, 4, 8, and 16 kHz tones), until 10 dB below the sound level with no visible ABR response. The threshold was defined as the interpolated sound level at which the ABR N1–P2 peak was 0.3 μV. Preoperative threshold dB peak equivalent SPL of <55 dB were considered to indicate normal hearing. See for further details Ramekers et al. (2014).

#### 2.3.2. Electrocochleography

Electrocochleography was performed using the gold-ball electrode as active electrode (situated in round window niche), a screw on the skull for reference electrode, and a needle in the left hindlimb muscle as ground electrode.

All recordings were performed in a sound-attenuated room. Stimuli were presented in a free-field 10 cm from the right pinna, using the same Bowers and Wilkens speaker as for the ABRs. The stimuli consisted of pure tone pips ranging from 0.25 to 16 kHz in octave steps, which were presented with alternating polarity and with an inter-stimulus interval of 99 ms.

Our stimulus parameters are chosen to be long enough to measure the CM, and to have sufficient rise and fall times to avoid spectral splatter. Therefore, we applied 2 or more periods of rise-fall time and 2 or more periods of plateau (Stronks et al., 2010; Havenith et al., 2013). Durations of the tones was 8 ms for the high frequencies (4–16 kHz) with rise/fall time of 1 ms. The 1 kHz and 2 kHz stimuli had duration of 8 ms, and rise/fall time of respectively 2 ms and 1.5 ms. The 500 Hz tone had a duration of 12 ms, with rise/fall time of 4 ms. And lastly, the 250 Hz tone had a duration of 24 ms with rise/fall time of 8 ms.

Sound levels were chosen sufficiently high to assess amplitudes and latencies at the same level at each stage, and with sufficiently small step sizes to assess the threshold. Stimuli were presented at maximum sound level, which differed across frequencies (in dB SPL): 99 at 250 and 500 Hz, 103 at 1 kHz, 98 at 2 kHz, 104 at 4 kHz, 110 at 8 kHz, and finally 107 at 16 kHz. The sound level was attenuated in 10 dB steps, starting with maximum sound level, until 10 dB below the sound level with no visible CAP and CM response.

The signal was pre-amplified using the same preamplifier as for the ABRs (amplification 5000x; band-pass filtered 1 Hz–30 kHz). The amplified signal was digitized by the same TDT3 system for analysis. The responses were averaged over 500 repetitions (maximum). Sometimes fewer repetitions were needed to achieve a reliable response, with typically smaller responses with low signal-to-noise ratio needing more repetitions.

#### 2.3.3. Tissue fixation and histological processing

After completing all ECochG measurements the animals were terminated with an overdose of pentobarbital injection intracardially. The right cochlea was then harvested for histological analysis. Intra-labyrinthine cochlear fixation was done with a fixative of 3% glutaraldehyde, 2% formaldehyde, 1% acrolein, and 2.5% dimethyl sulfoxide (DMSO) in a 0.08 M sodium cacodylate buffer, as described by a previous study (de Groot et al., 1987). The cochleas were decalcified in 10% EDTA for around 10 days, secondarily fixed in 1% osmium tetroxide and 1% potassium ruthenium cyanide, and embedded in Spurr’s low-viscosity resin. Staining was done with 1% methylene blue, 1% azur B, and 1% borax in distilled water. Tissue was sectioned using LeicaRM2265 microtome. From each cochlea, 5 midmodiolar sections of 1 μm were obtained in sequential manner and put on a slide with coverslip.

### 2.4. Data analysis

#### 2.4.1. Histology

**FIGURE 2:**
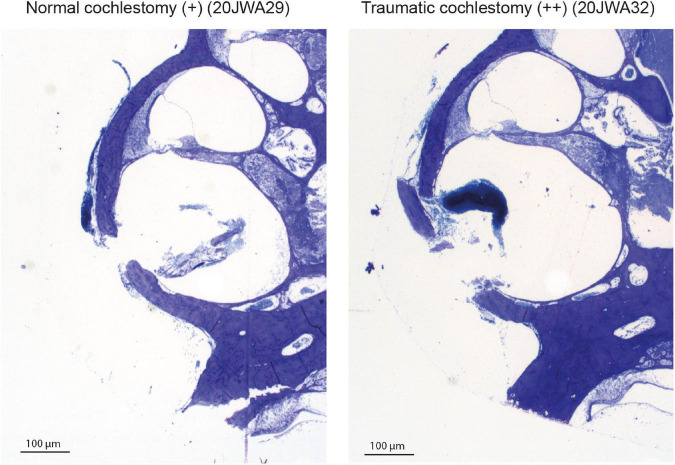
Two examples of the cochleostomy site: 20JWA29 shows a normal cochleostomy site, with no additional fracture of the lateral wall besides the intended cochleostomy. 20JWA32 shows a traumatic cochleostomy with an additional fracture of the lateral wall, splintered bone, and red blood cells near the cochleostomy.

Macroscopic cochlear trauma was assessed with light microscopy in standardized midmodiolar sections. The following items were used for trauma severity rating: fracture of modiolar wall (yes or no), OSL fracture (yes or no), and lateral wall damage around cochleostomy (as expected: +, more traumatic: ++). A more traumatic cochleostomy is considered to have fractured the lateral wall at different place than the site of cochleostomy (see Figure 2). Additional features of traumatic cochleostomy are more red blood cells, and splintered fragments of bone. In addition, the midmodiolar sections were used for quantification of inner and outer hair cells from base to apex, and the structural integrity of these hair cells was evaluated. Animals with affected structural integrity of inner or outer hair cells (e.g., dislocated or abnormally shaped), or loss of these hair cells were rated in general as having hair cell damage (yes or no).

#### 2.4.2. Electrocochleography

**FIGURE 3:**
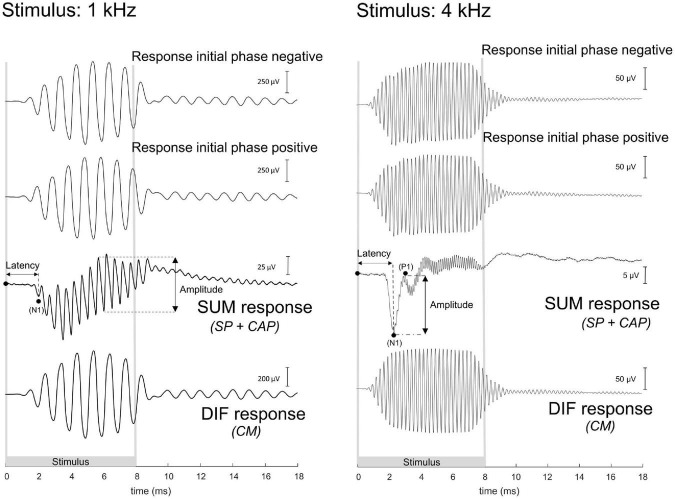
These are example electrocochleography responses using the gold-ball electrode, before cochleostomy in the same animal (20JWA29), to a low and high frequency tone. Duration of the stimulus differed between the different frequencies, and the stimuli were presented in alternating polarity. The compound action potential (CAP) is derived from the SUM response by adding the two initial responses together. At frequencies between 0.25 and 1 kHz, in this instance at 1 kHz, the CAP latency is derived from the N1 peak, and the CAP amplitude was derived from the largest peak-to-peak amplitude of the ongoing response. At the other frequencies of 2–16 kHz, in this instance at 4 kHz, the CAP latency was similarly extracted with the N1 peak, and the CAP amplitude as the N1P1 peak-to-peak at the onset of the response. The cochlear microphonics (CM) is derived from the difference (DIF) response by subtracting the initial responses from each other. For high stimulation levels the SUM response will contain some CM in addition to the CAP since the CM at those levels may not be symmetric (Fontenot et al., 2017). For low frequencies the DIF response will contain some neural responses in addition to CM since the phase-following responses to opposite polarity have opposite phase. Note: the late responses after stimulus offset may be evoked by echoes, 20–30 dB below the actual stimulus level, caused by reflections of the experimental chamber. The 1 kHz tone was presented at 103 dB SPL and the 4 kHz tone at 84 dB SPL.

**FIGURE 4:**
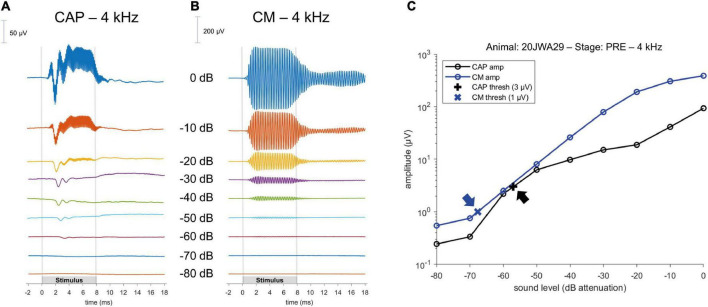
Example of a CAP; **(A)** and a cochlear microphonics (CM; **B**) response to a 4 kHz tone across different sound levels. Stimulus started with maximum sound level (in this instance of 4 kHz, at 104 dB SPL) and was attenuated in 10 dB steps until the CAP or CM response was not visible anymore. Subsequently, an input/output curve was constructed, and the threshold was interpolated using pre-defined threshold criteria **(C)**; arrows point to the thresholds.

The ECochG was analyzed using custom-written MATLAB scripts. See Figure 3 for example ECochG, with analysis of the response to one low-frequency (1 kHz) and one high-frequency (4 kHz) tone. To extract the CAP and the cochlear microphonics (CM), alternating polarity stimulation (condensation-leading and rarefaction-leading) was used. The CAP was analyzed by summation of the two responses, i.e., the SUM response. For frequencies 2–16 kHz, the N1-P1 peak-to-peak amplitude was determined. For the other frequencies ranging between 0.25 and 1 kHz, because of the ongoing auditory nerve response (also known as auditory nerve neurophonic), the largest peak-to-peak amplitude was determined, which was not always at the start of the response. Note that we simply refer to the nerve responses at low frequencies as CAP rather than neurophonic, as each peak represents a sum of action potentials. The amplitudes vary among animals as it depends on electrode positions of both the gold-ball and reference. Therefore, we examined the change of amplitude relative to the PRE stage by computing the ratio (POST/PRE). The CAP threshold criterion was an amplitude of 3 μV for high frequencies (4–16 kHz), and an amplitude of 1 μV for low frequencies (0.25–2 kHz). Thresholds were assessed by interpolation of the two data points around threshold (one above, one below). In case no data were acquired below threshold, we applied extrapolation of the data points at the lowest two sound levels. Threshold shifts (in dB) per POST stage, were determined with PRE values as reference for statistics, unless stated otherwise. The latency assessment was based on the N1 peak for all frequencies, and again shifts per POST stage (in ms), with PRE as reference, were used for analysis. Figure 4A shows an example of CAP for a 4 kHz tone at different attenuated sound levels, starting at maximal sound level, and an I/O curve is obtained to derive the interpolated threshold (Figure 4C). These recordings were performed before cochleostomy (PRE stage).

To analyze the CM the two responses (condensation-leading and rarefaction-leading) were subtracted, i.e., the DIF response. The CM threshold criterion was 1 μV for all frequencies. However, speaker artifacts were also present during measurements, which was evident in the click-evoked responses as an isolated peak at stimulus onset. Based on the magnitude of that peak, thresholds of the speaker artifacts were obtained for every measurement. Only CM data were included in our analyses that were larger than artifact. Figure 4B shows an example of CM for a 4 kHz tone at different attenuated sound levels, starting at maximal sound level, and an I/O curve is obtained to derive the interpolated threshold (Figure 4C).

### 2.5. Statistics

Threshold shifts are described as means with standard deviation. Repeated measures ANOVA was used to test for effects of independent variables (trauma group, stage, and frequency) on CAP threshold shifts, amplitude ratios and latency shifts. In the ANOVA shifts were based on PRE values. Pairwise comparisons were performed for the stages after ANOVA, with Bonferroni corrections. Correlation between CAP amplitudes and thresholds, and CAP and CM thresholds were tested using non-parametric Spearman’s rank correlation. *P*-values were based on two-tailed tests, and *p* < 0.05 was considered as significant.

## 3. Results

### 3.1. Animals

All animals had normal preoperative click-evoked ABR thresholds (mean 43 dB peSPL, range 36–50). Two out of 13 animals were excluded because their PRE stage CAP threshold values were ∼20 dB more than the average threshold on at least 2 out of 7 tested frequencies. The standard deviation of the mean CAP threshold of the remaining 11 animals at every separate frequency (0.25–16 kHz, octave steps) was ∼5 dB (range: 4.5–5.6 dB).

### 3.2. Histology

**FIGURE 5:**
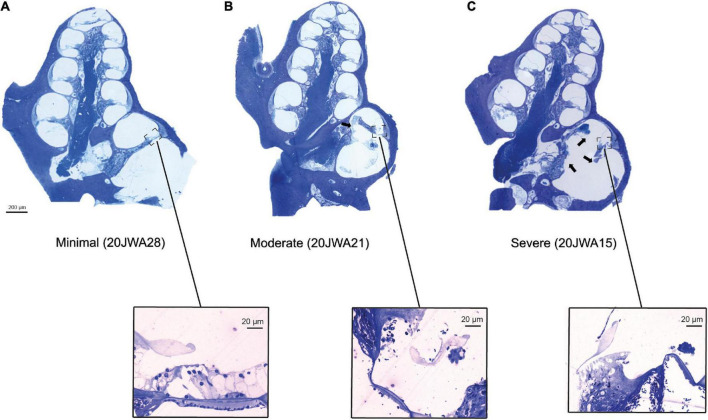
Three midmodiolar section examples (2.5x, light microscopy) are shown of each trauma group. In the minimal trauma group **(A)**, no other trauma than the cochleostomy was observed. In the moderate trauma group **(B)**, a fracture of the osseous spiral lamina is clearly seen in the basal turn (arrow). In the severe trauma group **(C)**, in addition to fracture of OSL, the modiolar wall is damaged at the basal turn. Additionally, the organ of Corti of the lower basal semiturn is shown at greater magnification (25x): **(A)** intact organ of Corti with 3 outer hair cells and one inner hair cell; **(B)** absent inner and outer hair cells; **(C)** absent inner and outer hair cells.

Midmodiolar sections were assessed for trauma. Based on these assessments, three groups of animals were identified: with minimal trauma (*n* = 3), moderate trauma (*n* = 5) or severe trauma (*n* = 3). See Figure 5 for one histological example for each trauma group, and Table 1 for an overview of the results of all animals. In addition to the cochleostomy, the severe group had OSL fracture and a fracture of the modiolar wall. Two of the moderately affected animals had more severe damage to the lateral wall near cochleostomy, and the other three animals had OSL fracture, but no fracture of the modiolar wall. In cases with trauma, it was always located at the site of cochleostomy and electrode insertion, i.e., at the basal turn of the cochlea.

**TABLE 1:** Trauma severity assessment based on histology.

Animal	Hair cell damage	OSL Fracture	Modiolar wall fracture	LW damage at cochleostomy	Trauma rating
20JWA15	+	+	+	++	severe
20JWA16	+	+	+	++	severe
20JWA17	+	+	+	++	severe
20JWA11	+	+	−	++	moderate
20JWA18	+	+	−	++	moderate
20JWA21	+	+	−	++	moderate
20JWA32	−	−	−	++	moderate
20JWA33	−	−	−	++	moderate
20JWA28	−	−	−	+	minimal
20JWA29	−	−	−	+	minimal
20JWA30	−	−	−	+	minimal

OSL, osseous spiral lamina; LW, lateral wall.

Additionally, both inner and outer hair cells were counted in the midmodiolar sections (see Table 2). All three cochlear turns were assessed, i.e., basal, middle and apical. The minimal trauma animals had no damage to either inner or outer hair cells across all three turns, except for one inner and outer hair cell that was absent more apically in 20JWA28. Hair cells were much more severely affected for both the moderate and severe trauma animals. In the moderate trauma group, one animal (20JWA11), had damage to both inner and outer hair cells at the basal turn, and loss of these cells at the middle and apical turn. Another moderately affected animal (20JWA21) also had basal damage to both inner and outer hair cells, and largely intact hair cells at the other turns. In addition, animal with moderate trauma (20JWA18) had a patchy loss of outer hair cells, i.e., some hair cells were missing in both the basal turn and middle turn, although the section was not entirely cut in the midmodiolar plane. The other two animals (20JWA32 and 20JWA33) had no damage or loss of any hair cells. Finally, in the severe trauma group, animal 20JWA15 and 20JWA16 had damaged inner and outer hair cells at all turns, and 20JWA17 only damaged of all inner and outer hair cells at the basal turn.

**TABLE 2:** Hair cell count in one midmodiolar section for every animal.

Animal number	IHC basal	OHC basal	IHC mid	OHC mid	IHC apex	OHC apex	Trauma severity
20JWA11	1	3	0	0	0	0	MO
20JWA15	0	0	0	0	0	0	SE
20JWA16	0	0	0	0	0	0	SE
20JWA17	0	0	2	6	2	6	SE
20JWA18	1	3	2	5	1	6	MO
20JWA21	1	3	2	5	2	6	MO
20JWA28	2	6	2	5	1	6	MI
20JWA29	2	6	2	6	2	6	MI
20JWA30	2	6	2	6	2	6	MI
20JWA32	2	6	2	6	2	6	MO
20JWA33	2	6	2	6	2	6	MO

IHC, inner hair cell; OHC, outer hair cell; MI, minimal trauma; MO, moderate trauma; SE, severe trauma; Basal, lower and upper basal semiturns; Mid, lower and upper middle semiturns; Apex, lower and upper apical semiturns. Two IHCs and six OHCs are present normally in normal-hearing guinea pigs for each of the three cochlear areas (basal, mid, and apex).

### 3.3. Electrocochleography

#### 3.3.1. Individual animals

**FIGURE 6:**
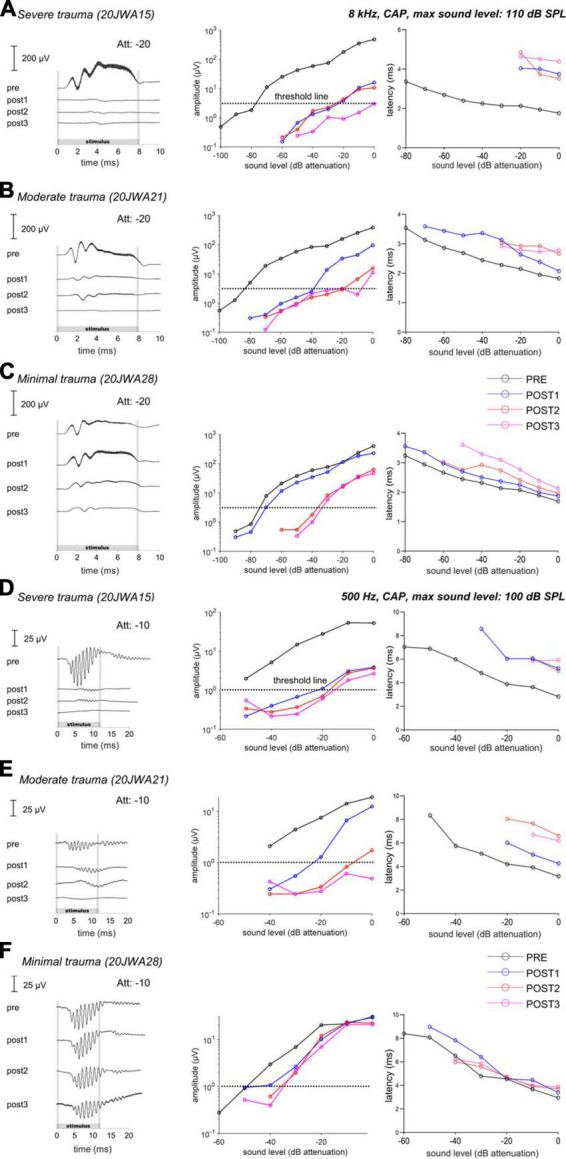
The CAP responses to an 8 kHz **(A–C)** and 500 Hz **(D–F)** tone of three individual animals is shown for all 4 stages. In the upper row **(A,D)**, the responses of a severely affected animal are shown. It is clear that after cochleostomy the responses were severely affected (high threshold and latency shifts), to both a high and low frequency tone. In the middle row **(B,E)**, the responses of a moderately affected animal are shown. In this animal, the responses are less severely affected, but still both responses to a low and high frequency were affected. In the lowest row **(C,F)**, the responses of a minimally affected animal are shown. In this animal, the responses to a high frequency tone are especially affected after electrode-array insertion. Att, attenuation levels.

To determine the relationship between ECochG and trauma severity we primarily analyzed the CAP. Figure 6 shows an overview of the trauma groups (based on histology), by providing an example of one individual SUM response to a high frequency (8 kHz) and low frequency (500 Hz) tone. It shows the responses for these three animals at ∼90 dB SPL across all 4 stages, and the respective CAP amplitudes and latencies as function of sound level. The PRE stage showed roughly equal CAP thresholds for these three animals at 8 kHz (range: 27–36 dB SPL, i.e., ∼80 dB attenuation) and at 500 Hz (range: 44–53 dB SPL, i.e., ∼50 dB attenuation).

At POST1, the CAP responses are in line with histological outcomes, i.e., the severe trauma animal (Figure 6A) having the smallest amplitudes (e.g., at ∼90 dB SPL: 5 μV) at 8 kHz; while the moderate trauma animal (Figure 6B) had larger responses (at ∼90 dB SPL: 30 μV), and the minimal trauma animal (Figure 6C) had the largest responses (at ∼90 dB SPL: 115 μV). The threshold shifts were 55 dB, 45 dB and 5 dB for the severe, moderate and minimal trauma animal respectively. Similarly, the CAP latency (at 90 dB SPL) increased most for the severe trauma animal with a latency shift of 2 ms, followed by 0.5 ms for the moderate trauma and 0.2 ms for the minimal trauma animal. In addition, not only higher frequencies were affected at POST1, but also lower as illustrated here for 500 Hz (see Figures 6D–F). CAP thresholds, amplitudes and latencies, showed similar results to 8 kHz tone, with smallest responses, largest threshold and latency shifts for the severe trauma animal (5 μV amplitude; 30 dB threshold shift and 2.6 ms latency shift), and largest responses and smallest threshold and latency shifts for the minimal trauma animal (25 μV amplitude; 5 dB threshold shift and 0.01 ms latency shift).

The POST2 and POST3 stages are described together, as they showed largely similar responses. At these two stages, the CAP threshold, amplitude and latency were similar to the POST1 responses for the severe trauma animal (Figures 6A, D). The moderate trauma animal had bigger differences between the CAP thresholds at POST2/POST3, than at POST1 (Figures 6B, E). The threshold and latency increased at POST2 for both 8 kHz and 500 Hz, and, albeit less, at POST3. For the minimal trauma animal, the CAP thresholds and latencies increased, of all the stage, the most at POST2, for both 8 and 0.5 kHz. At POST3, the responses were mostly similar to POST2 for the minimal trauma animal, except for latencies.

#### 3.3.2. Groups

##### 3.3.2.1. CAP threshold

**FIGURE 7:**
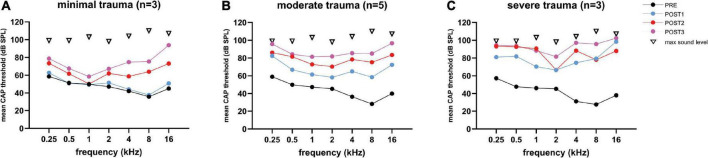
The mean CAP thresholds of all three trauma groups are shown across all tested frequencies (0.25–16 kHz), and the 4 stages. The PRE values were comparable between the three groups. Threshold shifts in all three groups were observed across all frequencies, and increased with every POST stage, except for the minimal trauma group **(A)**, in which barely any threshold shift was observed across all tested frequencies at POST1. The ranges among animals vary considerably between frequencies: **(A)** for minimal trauma 1 to 27 dB for low frequencies (0.25 to 2 kHz), and 4 to 52 dB for high frequencies (4 to 16 kHz); **(B)** for moderate trauma 8 to 50 dB for low frequencies, and 8 to 70 dB for high frequencies; **(C)** for severe trauma 5 to 30 dB for low frequencies, and 4 to 33 dB for high frequencies.

Figure 7 shows the mean CAP thresholds (in dB SPL) were plotted for each of the three trauma groups across for all tested frequencies (range: 0.25–16 kHz). The largest threshold increases occurred at POST1 for the severe group, with a mean of 37 dB increase (across all frequencies), followed by 23 dB for the moderate group, and just 4 dB for the minimal group. A difference in CAP thresholds was observed between the higher frequencies (4–16 kHz), and lower frequencies (0.25–2 kHz). The mean shift of the higher frequencies was higher with 43 dB (SD: 9), 28 dB (SD: 14) and 3 dB (SD: 4) for respectively the severe, moderate and minimal group at POST1. The lower frequencies had mean shifts of 26 dB (SD: 9), 17 dB (SD: 8) and 1 dB (SD: 4) for respectively the severe, moderate and minimal group at POST1.

At POST2, the minimal group had the largest threshold increase regarding the higher frequencies, i.e., additional shift of 27 dB (SD: 16), while the moderate group had an additional shift of 14 dB (SD: 21), and the severe group an additional shift of 14 dB (SD: 13). The additional threshold shifts for the lower frequencies were comparable between the groups, i.e., 11 dB (SD: 14), 10 dB (SD: 13) and 9 dB (SD: 10) for respectively the severe, moderate, and minimal group. At POST3, the additional threshold shift of ∼20 dB increase for the higher frequencies was comparable for all three groups. The thresholds at the lower frequencies increased at POST3 less than the higher frequencies, ∼7 dB.

Repeated measures ANOVA confirms that frequency, stage and trauma group all had a significant effect on CAP threshold shifts (see Table 3), with respectively *p*-values of <0.0001, <0.001, and 0.006. In addition, no interaction effects were observed between frequency, stage and trauma group for all possible combinations (*p* > 0.2). Lastly, *post hoc* analysis showed that between pairs of stages the CAP threshold shifts were significantly different between all combinations (i.e., POST1 vs. POST2, *p* = 0.007; POST1 vs. POST3, *p* < 0.001; POST2 vs. POST3, *p* = 0.002).

**TABLE 3:** Results from the repeated measures ANOVAs.

CAP		Main effects				Interaction effects		Between pairs analysis^3^		
		Frequency^1^	Stage	Group	Freq × Stage^1^	Freq × Group^1^	Stage × Group	Post^1^ vs. Post^2^	Post^1^ vs. Post^3^	Post^2^ vs. Post^3^
Threshold	*F*	15.86 (2.60)	38.10 (2.00)	10.47 (2.00)	1.36 (4.11)	1.12 (5.21)	0.71 (4.00)	−	−	−
	*P*	<0.001	<0.001	0.006	0.27	0.38	0.60	0.007	<0.001	0.002
Amplitude	*F*	10.34 (3.26)	21.95 (2)	14.33 (2.00)	1.15 (3.96)	1.94 (6.53)	1.04 (4)	−	−	−
	*P*	<0.001	<0.001	0.002	0.35	0.11	0.42	0.22	0.01	0.004
Latency	*F*	16.72 (1.21)	5.29 (1.21)	7.79 (2.00)	3.53 (2.13)	0.79 (2.42)	0.90 (2.41)	−	−	−
	*P*	0.002	0.040	0.013	0.05	0.51	0.46	0.06	0.03	0.03

^1^Greenhouse-Geisser corrected.

^2^Interaction of Freq × stage × group was not significant for all three variables.

^3^Bonferroni correction.

Degrees of freedom (*df*) given in bracket.

##### 3.3.2.2. CAP amplitude

**FIGURE 8:**
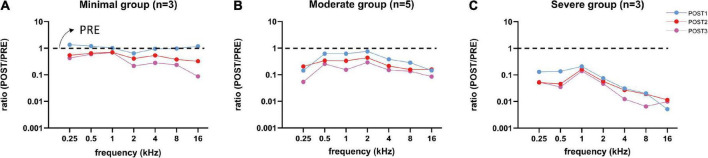
Mean CAP amplitude ratios (POST/PRE) at ∼90 dB SPL of all three trauma groups are shown for each stage. After each stage, the amplitudes decreased for all three groups, except at POST1 stage in the minimal trauma group **(A)**. Responses of the moderate group **(B)** were affected more than in the minimal group and less than in the severe group. Responses to high frequency tones were severely affected, however also responses to low frequency tones were affected, especially in the severe trauma group **(C)**.

We examined the change of amplitudes relative to the PRE stage by computing the amplitude ratio. Figure 8 shows the log10 of the mean amplitude ratios at ∼90 dB SPL for POST1-3 stages for every trauma group. Across all frequencies the CAP amplitude decreased with each consecutive surgical procedure. As expected, high frequencies (4–16 kHz) were affected, however also low frequencies (0.25–2 kHz), e.g., at 16 kHz, the mean amplitude ratio of all animals at POST1 was 0.44, and at 500 Hz the mean ratio was 0.65. Every subsequent stage had lower amplitude ratios. Repeated measures ANOVA shows that frequency, stage and trauma group all had a significant effect on CAP amplitude (see Table 3), with *p*-values of <0.001, <0.001, and 0.002, respectively. No interaction effects were observed between frequency, stage and trauma group for all possible combinations (*p* > 0.1).

**FIGURE 9:**
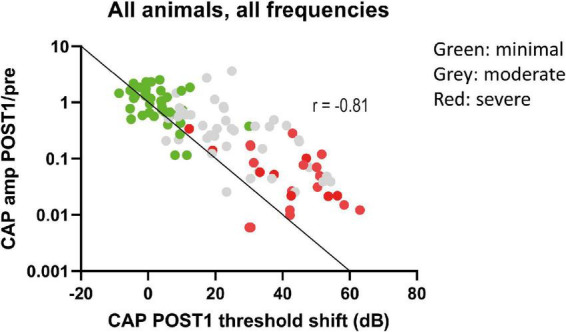
The CAP amplitude ratios (POST1/PRE) at ∼90 dB SPL were plotted against CAP threshold shifts at POST1. Data points represent all individual responses of all animals to the tested frequencies (0.25–16 kHz, octave steps). The data points are dispersed according to trauma group, with minimal trauma animals having less decline of amplitude and threshold shift increase. The line represents the 1 dB amplitude decrease with 1 dB threshold shift. A decrease of these amplitudes at 90 dB SPL was strongly correlated with threshold shifts (Spearman’s *r* = –0.81, *p* < 0.0001).

We analyzed the correlation between CAP threshold shifts and CAP amplitudes ratio (at 90 dB SPL) at POST1 for all frequencies (Figure 9). In general, as shown before, the amplitude ratio values and thresholds are corresponding to histology grouping (i.e., minimal, moderate or severe trauma), with the minimal group animals having largely the same amplitude as in the PRE stage, with small threshold shifts, and the severely affected animals having amplitude magnitudes of around 1% of the PRE stage amplitudes, and thresholds shifts of ∼60 dB. The increased threshold and decline in amplitudes at POST1 of all three groups was correlated (Spearman’s *r* = 0.81, *p* < 0.0001). Analysis of the trauma groups separately also showed significant correlation for minimal group (Spearman’s *r* = 0.47, *p* = 0.033), for moderate group (Spearman’s *r* = 0.55, *p* = 0.001) and for severe group (Spearman’s *r* = 0.47, *p* = 0.029). Lastly, *post hoc* analysis showed that between pairs of stages the CAP amplitude shifts were significantly different between POST1 vs. POST3, *p* = 0.01; POST2 vs. POST3, *p* = 0.004, however not for POST1 vs. POST2 (*p* > 0.2).

##### 3.3.2.3. CAP latency

**FIGURE 10:**
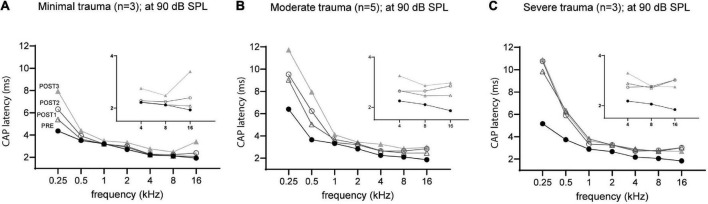
The mean CAP latencies at 90 dB SPL of the three trauma groups are shown for all stages, with insert graphs highlighting the differences at the higher frequencies (4–16 kHz). The PRE values were comparable between the three groups. In addition to high frequencies (4–16 kHz), the lower frequencies (0.5–2 kHz) were also affected in all three groups. The minimal trauma group had the lowest latency shifts **(A)**, followed by the moderate trauma group **(B)**, and the highest shifts were observed for the severe trauma group **(C)**.

The mean latency at ∼90 dB SPL for each group across all tested frequencies was plotted in Figure 10. At each stage, the latency was dependent on trauma severity, with more trauma leading to longer latencies. The CAP latencies for all frequencies were affected by the surgical interventions, but the degree differed between the lowest frequencies (0.25 and 0.5 kHz) and the higher frequencies (1–16 kHz). This latter finding is due to the much longer period lengths of the lower frequencies (period = 1/f). The severe trauma group reached maximum latencies at POST1 (i.e., mean of 10 ms and 6 ms for 0.25 and 0.5 kHz; and mean ∼3 ms for the higher frequencies). In contrast, the minimal trauma animals showed a gradual increase of latency with each consecutive stage. The low frequencies’ mean latency increased starting with 3.9 ms in PRE stage, to 4.5 ms at POST1, 5.1 ms at POST2 and finally 6.1 ms for POST3. The higher frequencies also showed a trend of latencies becoming longer, with mean latency from 2.3 ms at PRE to 2.5 ms at POST1, 2.7 ms at POST2, and 3.0 ms for POST3. The moderate trauma group showed similar to the mild group gradual increases with each stage, but with larger steps. Repeated measures ANOVA showed that frequency, stage and trauma group all had a significant effect on CAP latency shift (see Table 3), with *p*-values of 0.002, 0.040 and 0.013 respectively. No interaction effects were observed between frequency, stage and trauma group for all possible combinations. Lastly, *post hoc* analysis showed that between pairs of stages the CAP latency shifts were significantly different between POST1 vs. POST3, *p* = 0.03; POST2 vs. POST3, *p* = 0.03, however not for POST1 vs. POST2 (*p* > 0.05).

#### 3.3.3. Cochlear microphonics

**FIGURE 11:**
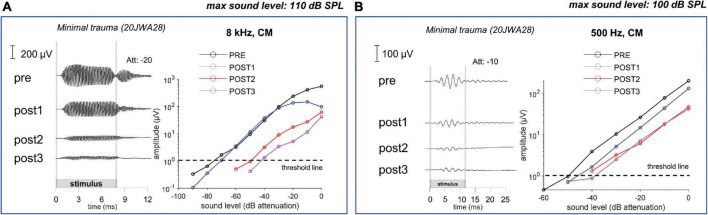
The cochlear microphonic responses to an 8 kHz **(A)** and 500 Hz **(B)** tone of one individual minimal trauma animal are shown for all 4 stages at ∼90 dB SPL. The graphs show the input/output curve, with threshold criterium of 1 μV amplitude (peak-to-peak). The highest threshold shifts are seen after electrode insertion (POST1) to an 8 kHz tone, however also threshold shifts occurred for the responses to a low frequency tone.

**FIGURE 12:**
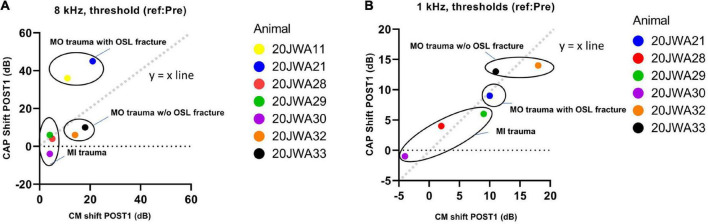
The correlation between the CAP shift and cochlear microphonics (CM) shift at POST1 stage was assessed. 20JWA11 CM threshold at 1 kHz was near the artifact threshold and is therefore omitted. The threshold shifts are shown for responses to 8 kHz **(A)** and 1 kHz **(B)** tone. Individual datapoints are shown. Animals with osseous spiral lamina (OSL) fracture had higher CAP threshold shifts than CM threshold shift at 8 kHz **(A)**. However, at 1 kHz, the CAP and CM threshold shift were strongly correlated, with Spearman’s *r* = 0.95, with *p* = 0.004.

In Figure 11 an example of CM responses (DIF) is shown for a sound level of ∼90 dB SPL for a 8 kHz tone (Figure 11A) and a 500 Hz tone (Figure 11B), and I/O curves for an animal with minimal trauma (animal 20JWA28). For both frequencies the CM was affected by the surgical phases. In addition, as was observed for the CAP responses, the CM responses declined the most at POST2 (i.e., after electrode insertion). In general, threshold shifts were similar for CAP and CM at POST1. Figure 12 shows two exceptions: two moderately affected animals with OSL fracture had much higher CAP threshold shifts (∼40 dB) than CM threshold shifts (∼18 dB) at 8 kHz. This effect was also seen at 4 and 16 kHz (not shown). In contrast, the remaining two animals in the moderate trauma group, without OSL fracture, had slightly more CM threshold increases (∼20 dB) than CAP threshold increases (∼15 dB). The animals with minimal trauma had the lowest CAP and CM threshold shifts, with approximately equally affected CAP and CM thresholds. At a lower frequency, at 1 kHz, the CAP and CM threshold shifts were highly correlated for both moderate and minimal group animals (Spearman’s *r* = 0.95, *p* = 0.004), without any outliers.

## 4. Discussion

Electrocochleography is a promising tool that might aid the surgeon in minimizing acute trauma during cochlear implantation. To understand the relationship between ECochG and the separate stages of cochlear implantation, we investigated primarily CAP thresholds, amplitudes and latencies before cochleostomy, after cochleostomy, after electrode-array insertion and after withdrawal of the electrode array. We show that ECochG can be affected by both cochleostomy and subsequent insertion of an array, and that these responses declined even further with withdrawal of the array. Additionally, basal trauma, inflicted by cochleostomy, array insertion and/or withdrawal of array, affected not only high frequency regions, but also more apical lower-frequency regions.

In general, the induced damage was more substantial than we had anticipated. In only 3 out of 11 cases threshold shifts were mild, and correspondingly histological damage in those cases was limited, and similar to the results of Andrade et al. (2022). In an acute ECochG study in guinea pigs they showed minimal effects of cochleostomy and thresholds shifts of around 20 dB after array insertion only for high frequencies mainly corresponding to the location of the electrode array. We think that the moderate and severe cases we observed may be typical for the clinical situation in which cochlear implantation surgery is performed. Our data show that without the explicit hearing preservation approach moderate to severe trauma leads to substantial loss of low-frequency hearing.

### 4.1. Cochleostomy

We should note that of the steps investigated, nowadays, cochleostomy is the less common approach for cochlear implantation (Kant et al., 2022b). However, cochleostomy and extended round window (which is a related approach as it also requires drilling) are expected still to be used in the future as these approaches are preferred in certain anatomical conditions and for perimodiolar electrode arrays (Garaycochea et al., 2020; Jwair et al., 2022).

The CAP changes after cochleostomy correlated with the severity of trauma that was inflicted at the basal turn. The largest CAP changes were probably cause by modiolar wall fracture. Less severe CAP changes occurred with OSL fracture, and almost no CAP changes occurred in the minimal trauma group. The only CAP changes in animals without structural trauma were minor latency increases. In animals with structural trauma, a threshold shift at high frequencies (4–16 kHz) was always accompanied with a threshold shift at low frequencies (0.25–2 kHz) that was 15 dB smaller. Apparently, (severe) local basal trauma due to the cochleostomy, can affect CAP responses of the apical turn.

The high-frequency loss in animals with trauma, both moderate and severe, might be related to OSL fracture at the basal turn that damaged the peripheral processes of the basal SGCs. In the severely affected animals, fracture of the modiolar wall at the basal turn, probably caused additional trauma to the cell bodies of the basal SGCs. The low-frequency loss can be related to the basal trauma to the basilar membrane, which potentially impacted its sensitivity and its passive contribution to the traveling wave (Nuttall and Dolan, 1996), hereby reducing the sensitivity at the apical turn.

Local basal trauma in guinea pigs can affect SGCs more than the basilar membrane in some instances (Figure 11). We observed that CAP threshold shifts were larger than CM threshold shifts at 8 kHz in animals with OSL fracture at the basal turn. This can be explained by the relatively large OSL, which crosses the space between the modiolus and the organ of Corti. Therefore, the OSL can be damaged without violating the borders of the scala media. In addition, considering the angle of the cochleostomy, the OSL was fractured near the modiolar side (more medial) rather than near the basilar membrane (i.e., more lateral). In a human study, it has been shown that, indeed, a change of position of the cochleostomy site, i.e., more anteriorly than inferiorly, might damage the OSL more easily (Iseli et al., 2014). If the discrepancy of CAP and CM thresholds at higher frequencies is indeed related to local trauma of the OSL, agrees with the CAP and CM thresholds being equally affected at a low frequency (Figure 11).

Chronic experiments in normal-hearing guinea pigs have shown that CAP responses to lower frequencies can be affected after array insertion (Honeder et al., 2016, 2019). Their cochleostomy position was similar to our study (0.5–1 mm from the RW in both studies), and similar CAP recordings were conducted with gold electrode wire on the RW (Honeder et al., 2018). However, because these studies measured only after insertion, the effect of cochleostomy is not entirely clear. In addition, those experiments were chronically performed, as opposed to our acute experiments. This raises important differences. Time point of measurement is delayed in the chronic experiments, raising the possibility of additional trauma that is not related to cochleostomy nor electrode insertion. Also, the (in)reversibility of the CAP responses could not be tested with withdrawal of array for obvious reasons in a chronically implanted animal. Still, these studies showed mean CAP threshold shifts of 20–30 dB for low frequencies (0.5–2 kHz) postoperatively after electrode insertion through a cochleostomy. These threshold shifts were similar to our study. We have shown in the present study that insertion trauma causes these changes, and in addition, that trauma due to cochleostomy might enhance these changes even more. The responses improved approximately 10 dB after 1 month in the Honeder studies, which raises the question whether the acute effects, as observed in our study, are temporarily. It is likely that the acute effects, that are caused by structural trauma, such as in our study with the moderate and severe group, are long lasting. In contrast, the threshold shifts in the minimal trauma group, even though threshold shifts occurred after array insertion, might be more of temporarily fashion. Progressive deterioration after cochleostomy affecting the cochlear responses is unlikely to have occurred in this study considering the short time intervals between the different stages (1–2 h), although it cannot be excluded.

### 4.2. Electrode array insertion

In our study, the electrode array reached approximately the upper basal semiturn of the cochlea in guinea pigs (depth of 4–5 mm). Based on the Greenwood function, the directly affected frequency range would be around 10–30 kHz (Greenwood, 1990; van Ruijven et al., 2005), which is near to or at our high frequency range of 4–16 kHz. Thus, changes to the CAP at the high frequencies are expected. However, in all trauma groups, electrode insertion trauma occurred also at low frequencies (threshold shift of 10 dB, ∼10 dB less than high frequencies).

Previous animal studies investigated mainly insertion trauma by measuring responses at the RW, and subsequently during electrode insertion through the RW. These studies were performed in normal-hearing, or high-frequency deafened gerbils, in which RW insertion is more feasible than in guinea pigs (Adunka et al., 2010; Choudhury et al., 2011; DeMason et al., 2012). In guinea pigs a pure round-window approach is not possible because of obstructing bony overhang. Without any drilling, it is impossible to guide the electrode array underneath this overhang, then upward into the round window niche, and then down again into the round window without damaging the electrodes. The studies with gerbils all showed higher CAP and CM thresholds for lower frequencies upon electrode insertion. Recordings performed with RW or intracochlear array electrodes, showed that the CAP and CM responses to low frequencies deteriorated after array insertion limited to the basal turn. These measurements correlated in most instances with anatomical damage to basilar membrane or OSL (or both).

Various factors might play a role in local basal turn trauma affecting the function of the apical cochlear areas. The array touching the basilar membrane changes its position, which affects the mechanical response. Other factors are reduction in blood flow volume, due to disruption of capillaries or small blood vessels around the basilar membrane and osseous spiral lamina, and additional disruption of the homeostasis when the stria vascularis is damaged (Nakashima et al., 2002; Shi, 2016). Red blood cells, which are ototoxic, at the basal region might be pushed more apically by the array during insertion. It may explain the hair cell loss in middle and apical turns in three cases, which must have occurred within 6 h and obviously contributed to functional loss (Table 2). Trauma to cochlear structures can lead to mixture of endolymph, located in the SM, and perilymph, which is located in the ST and SV, abolishing the endocochlear potential (Reiss et al., 2015). It has also been reported that intracochlear shift of pressures upon cochleostomy and/or array insertion can affect the general function of the cochlea acutely (Greene et al., 2016; Gonzalez et al., 2020). The array with its diameter of 0.5 mm is inserted for 4 mm into the scala tympani, reaching the location where the scala tympani has a width of about 0.5 mm (Wysocki and Sharifi, 2005). This may have caused increased pressure changing the basilar membrane position. Conversely, leakage of perilymph may reduce the pressure, thereby also negatively affecting the basilar membrane response (Todt et al., 2017). It is likely that the above-mentioned mechanisms all contribute to the (negative) effect of basal trauma on apical cochlear areas.

The damage is correlated to the size and stiffness of the electrode array. Studies have shown that lower threshold shifts occurred, at both low and high frequencies, with smaller and flexible arrays (Choudhury et al., 2014; Drouillard et al., 2017). Similar to the present study, insertion trauma was not always accompanied with (severe) structural trauma in those studies.

Interestingly, a study using ABR recordings in normal-hearing guinea pigs showed that at 1 week after cochlear implantation no difference in ABR threshold shifts was found between animals with and animals without OSL fracture at 2–32 kHz (Chambers et al., 2019). They did find increased thresholds of 20–30 dB in general. This discrepancy with our study might be due to methodological differences. The ABRs might be less sensitive to trauma, compared to CAP, since they are much smaller. In addition, they measured one week after surgery, which is possibly enough time for the non-trauma group to develop other traumas related to chronic implantation (e.g., tissue inflammation).

Withdrawal of the array led to a further worsening of responses, which indicates that trauma caused by insertion and removal, rather than the mere presence of the array, affects the responses. We reason that the presence of the array can affect the ECochG temporarily by touching the basilar membrane and/or increasing the scalar pressure. It can affect ECochG permanently by damaging structures. In the former case removal might have caused recovery of responses. On the other hand, removal may cause additional mechanical trauma and/or loss of perilymph. It has been shown that the endocochlear potential might be affected by presence of the electrode array (Oshima et al., 2014; McClellan et al., 2021). However, those studies also suggest that the role of the endocochlear potential is delayed after cochlear implantation, and might affect predominantly the area with direct trauma. It is therefore likely that the increased thresholds observed in this acutely performed study were not caused by a reduced endocochlear potential.

## 5. Conclusion

We found that cochleostomy can be performed without causing cochlear structural damage and effect on the responses, but that subsequent insertion leads to deterioration of the responses for each of the included 11 animals. The extent of deterioration of ECochG was associated with the severity of trauma by cochleostomy or electrode insertion. In addition, even though the cochleostomy is drilled in the basal turn and the electrode array does not reach beyond the basal turn, ECochG responses to the lower frequencies can be significantly affected as well. This implies that basal trauma should be minimized in order to preserve the low-frequency residual hearing of CI recipients. Even though minimal invasive procedures are conducted, the surgeon should be aware of the negative impact of this surgical procedure.

## Data availability statement

The raw data supporting the conclusions of this article will be made available by the authors, without undue reservation.

## Ethics statement

The animal study was reviewed and approved by the Animal Experiments Committee of Utrecht University and the Central Authority for Scientific Procedures on Animals.

## Author contributions

SJ performed the experiments and statistical analysis, organized the database, and wrote the first draft of the manuscript. All authors conceived and designed the study and contributed to the manuscript revision, read, and approved the submitted version.
